# BMI-based nutritional assessment of children aged 11–17 years in rural Ellisras, Limpopo province

**DOI:** 10.4102/jphia.v16i1.1295

**Published:** 2025-06-05

**Authors:** Themba T. Sigudu, Thandiwe N. Mkhatshwa, Kotsedi D. Monyeki

**Affiliations:** 1 Department of Physiology and Environmental Health, Faculty of Science and Agriculture, University of Limpopo, Sovenga, South Africa; 2 Department of Health and Society, Faculty of Health Sciences, University of the Witwatersrand, Johannesburg, South Africa

**Keywords:** anthropometric characteristics, obesity, underweight, adolescence, rural South Africa, public health

## Abstract

**Background:**

Childhood malnutrition, including both undernutrition and overnutrition, remains a significant public health concern in many low- and middle-income countries, including South Africa. Understanding the anthropometric characteristics and obesity predictors among rural South African adolescents is crucial for informing targeted public health interventions.

**Aim:**

This study aimed to assess the nutritional status of children in rural Ellisras, South Africa, aged 11–17 years, and to identify predictors of obesity.

**Setting:**

The study was conducted in Ellisras, a rural area in South Africa, between January 2021 and December 2021.

**Methods:**

A total of 1217 adolescents (612 boys, 605 girls) participated in the study. Anthropometric measurements, including height, weight and body mass index (BMI), were collected. Multivariate regression analysis was used to identify factors associated with obesity.

**Results:**

The findings indicated that 43.10% of children were classified as underweight, with a higher prevalence among boys (64.57%) than girls (35.43%). The overall prevalence of overweight and obesity was low (0.41%), with girls (80%) being more affected than boys (20%). Boys had a slightly higher average height (154.79 cm) than girls (154.60 cm), while girls had a higher average BMI (16.41 kg/m^2^) compared to boys (15.81 kg/m^2^). Multivariate regression analysis revealed that adolescents aged 15–16 years had significantly higher odds of obesity (adjusted odds ratio [AOR] = 2.10, *p* < 0.001) compared to 11–12-year-olds. Additionally, girls had significantly higher odds of obesity than boys (AOR = 2.80, *p* < 0.001).

**Conclusion:**

The study highlights the dual burden of malnutrition among rural South African adolescents, with a high prevalence of underweight and emerging obesity, particularly among girls.

**Contribution:**

These findings emphasise the need for targeted nutritional interventions, with special attention to adolescents aged 15–16 years and girls, who are at a higher risk of obesity.

## Introduction

Growth is a complex biological process influenced by both genetic and environmental factors.^1^ While certain determinants such as age, gender, genetics and developmental influences are beyond an individual’s control, other factors – particularly lifestyle-related variables like physical activity, nutrition and socio-environmental conditions – are modifiable.^2^ Nutritional status, which reflects the body’s condition because of nutrient intake, absorption and utilisation, plays a key role in physical development and overall health.^3^ The idea that adequate nutrition is a fundamental human right is crucial for ensuring the well-being of children, as it supports optimal physical, emotional and intellectual growth.^4^ For children aged 3–10 years, particularly in rural South African communities, proper nutrition is not only essential for immediate health but also has long-term implications for development.^5^

The Ellisras Growth Study highlighted the growth and nutritional status of rural South African children during 1999.^6^ The study showed that many children in these communities were at risk of undernutrition, which could impede their growth and cognitive development. This highlights the importance of addressing nutritional deficits early in life, as this critical period is vital for shaping both physical and cognitive growth. Incorporating adequate nutrition policies and interventions, particularly for children in underserved areas, is necessary to ensure that all children have the opportunity to thrive physically, emotionally and intellectually.

In South Africa, malnutrition – both acute and chronic – remains a significant public health concern. The country faces a double burden of malnutrition, with both undernutrition (e.g. stunting) and overnutrition (e.g. obesity) contributing to adverse health outcomes, particularly among children. Malnutrition is associated with one-third of all child in-hospital deaths, and approximately 27% of children under the age of five are stunted, while 12% are overweight.^7^ Alarmingly, both stunting and obesity prevalence are on the rise, creating a complex challenge for public health interventions.

Recent studies on adolescent nutrition in South Africa have highlighted the vulnerability of urban-dwelling girls to obesity. Dietary patterns are increasingly characterised by a shift towards energy-dense, processed foods high in sugar and fat but lacking in essential micronutrients.^8^ Furthermore, a range of factors, including family dynamics, peer influence and the broader food environment, shape adolescent food choices. Data from the South African Demographic and Health Survey (SADHS) indicate that 34% of girls and 17.2% of boys aged 15 years and older are anaemic, with a concerning prevalence of unhealthy dietary behaviours, such as regular consumption of salty snacks and low intake of fruits and vegetables.^9^

Among adolescents, autonomy over food choices increases, leading to behaviours such as frequent consumption of fast food, sweetened beverages and snacks, often purchased from school tuckshops.^10^ These dietary habits are concerning as they contribute to increased intake of salt, sugar and unhealthy fats, with significant implications for weight gain and the development of obesity. Studies have shown that the prevalence of overweight and obesity is higher among adolescent girls compared to boys, and this trend worsens with age.^11^ Rapid weight gain in childhood has been linked to early onset of puberty, further elevating the risk for obesity later in life.^12^ Notably, perceptions of body size, influenced by cultural and societal norms, may also contribute to unhealthy eating behaviours and an increased risk of developing eating disorders such as anorexia and bulimia.^13^

In essence, this study further supports the notion that adequate nutrition, which is crucial for growth and development, must be carefully balanced to avoid both undernutrition and overnutrition. Addressing dietary patterns and ensuring a balanced intake are crucial for fostering optimal health outcomes in children, especially those in rural or underserved communities.

The consequences of childhood obesity extend into adulthood, with increasing rates of overweight and obesity contributing to the growing burden of non-communicable diseases (NCDs) such as hypertension, diabetes and cardiovascular diseases. Among females, obesity is associated with poor pregnancy outcomes, including higher risks of preterm birth, low birth weight and maternal mortality.^14^ Given that adolescence represents a critical period for the prevention of nutrition-related NCDs, there is an urgent need for targeted interventions. However, data on adolescent nutritional status, particularly in rural settings, remain sparse.

The aim of this study was to assess the nutritional status of children residing in rural areas of Ellisras, using body mass index (BMI) cut-off points from the Centres for Disease Control and Prevention (CDC) reference standard, between March 2003 and April 2003. Additionally, this study sought to identify key factors associated with overweight and obesity within this population. Given the scarcity of recent data on rural adolescents in South Africa, this research aims to contribute valuable insights into the nutritional challenges faced by specific population groups, with a focus on demographic variables and regional differences.

## Research methods and design

### Study design and study site

This study utilised a cross-sectional design and was conducted as part of the ongoing Ellisras Longitudinal Study (ELS). Ellisras, now known as Lephalale, is located in the north-western part of the Waterberg District Municipality, Limpopo province, South Africa, situated between 23˚30’ and 24˚00’ South latitude and 27˚30’ and 28˚00’ East longitude. The municipality borders four other local municipalities – Blouberg, Modimolle, Mogalakwena and Thabazimbi – and is located near the international border between South Africa and Botswana. The rural areas of Ellisras encompass 42 settlements with an estimated population of approximately 50 000 inhabitants and are situated about 70 kilometres from Ellisras town.

The local economy is predominantly driven by mining, which contributes 59.21% to the gross domestic product (GDP) of the area. Other significant sectors include agriculture, manufacturing and electricity generation, with the latter accounting for 11.33% of the GDP and representing 69.65% of the Waterberg electricity sector.^15^ A considerable portion of the workforce engages in subsistence farming and cattle rearing, while a smaller group is employed in education and civil service. Despite these contributions, the region faces high levels of unemployment, poverty and low life expectancy, factors that significantly impact the rural communities in Ellisras.^16^

### Data source and data extraction

Data for this study were sourced from cross-sectional design records collected as part of the ongoing ELS. The sample comprised 1217 children, aged 11–17 years, from rural areas of Ellisras, including 612 boys and 605 girls. These data were compared with the CDC cut-off values for BMI to classify the weight status of participants into four categories: underweight, overweight and obesity.

For each child, demographic information and anthropometric measurements (weight, height and BMI) were extracted. These data provided a comprehensive overview of the children’s nutritional status, enabling the characterisation of the weight status distribution within this population.

### Data management and data analysis

Prior to analysis, data cleaning was conducted using Microsoft Excel to identify and address any inconsistencies, such as missing information or duplicate entries. No duplicate records were found for individual participants. The following variables were extracted for each child: survey date, age, sex, height and weight measurements.

Anthropometric data collection for all participants included measurements of weight and height. Weight was measured using an electronic scale with a precision of 0.1 kg, and height was measured with a Martin anthropometer to the nearest 0.1 cm. These measurements were then used to calculate each child’s BMI. As BMI for children and adolescents is age- and sex-specific, BMI-for-age was used in this study. Body mass index was computed using the following formula: weight (kg)/height^2^ (m^2^). Body mass index cut-off points from the CDC reference standard for children aged 11–17 years were applied to classify participants into weight status categories as follows: underweight (BMI ≤ 17.5 kg/m^2^), overweight (BMI ≥ 25.0 kg/m^2^) and obesity (BMI ≥ 30.0 kg/m^2^). The variable ‘age group’ was recategorised into distinct age group categories using the cohort-component method for population estimation produced by Statistics South Africa.

Data analysis was performed using Stata Statistical Software (Release 13; StataCorp LP, College Station, Texas, United States). Descriptive statistics were used to summarise categorical variables, which were reported as frequencies and percentages, and continuous variables, which were presented as means and standard deviations. The Student’s *t*-test was used to compare the means of continuous variables, while the Chi-squared test was applied to assess the differences in proportions of categorical variables.

A multivariate logistic regression analysis was performed to identify significant factors associated with obesity risk, with a focus on age and sex as predictors. These variables were selected based on a bivariate analysis, which revealed a significant association with weight status (*p* ≤ 0.2). A forward stepwise selection method was employed to construct the final model, and statistical significance was defined as *p* < 0.05.

### Ethical considerations

Ethical clearance to conduct this study was obtained from the University of Limpopo Turfloop Research Ethics Committee (No. TREC/323/2017:IR). The study on the nutritional status of children aged 11–17 years in rural Ellisras, Limpopo, followed strict ethical guidelines to ensure the protection and dignity of participants. Informed consent was obtained from parents, and children provided assent, with participation being voluntary. Participants’ privacy was safeguarded through anonymised data storage. The study was culturally sensitive, minimised risks and aimed to benefit the community by improving child nutrition. Local stakeholders were involved throughout the research process to ensure relevance and fairness, and findings were shared equitably. The study maintained transparency, avoided bias and disclosed any potential conflicts of interest to uphold integrity and accountability.

## Results

### Descriptive statistics

#### Age distribution of study participants

The demographic characteristics of the study participants are presented in Table 1. A total of 1217 child records from 42 rural settlements in Ellisras were included in the present study, comprising 50.29% (*n* = 612) boys and 49.71% (*n* = 605) girls. According to the survey records, the ages of the children ranged from 11 years to 17 years, with a mean age of 13 ± 1.44 years.

**TABLE 1:** Distribution of demographic characteristics of children living in rural areas of Ellisras according to age and sex, between March 2003 and April 2003, Limpopo province.

Age (years)	Boys (*n* = 612)			Girls (*n* = 605)		
	*n*	%	95% CI	*n*	%	95% CI
11	85	13.89	0.11–0.17	77	12.73	0.10–0.15
12	104	16.99	0.14–0.20	98	16.2	0.13–0.20
13	149	24.35	0.20–0.28	129	21.32	0.17–0.25
14	141	23.04	0.19–0.27	152	25.12	0.21–0.29
15	96	15.69	0.13–0.19	118	19.5	0.16–0.23
16	34	5.56	0.04–0.07	30	4.96	0.04–0.06
17	3	0.49	0.00–0.01	1	0.17	0.00–0.00

CI, confidence interval.

Among boys, the most prevalent age group was 13 years, comprising 24.35% (*n* = 149) of the sample. For girls, the highest proportion was found in the 14-year-old group, which constituted 25.12% (*n* = 152) of the female participants. For boys, the second most prevalent age group was 14 years, comprising 23.04% (*n* = 141) of the sample, while for girls, the 13-year-old group followed closely at 21.32% (*n* = 129). This was followed by 12-year-olds at 16.99% (*n* = 104) among boys and 15-year-olds, who accounted for 19.50% (*n* = 118) of the girls. The 15-year-olds made up 15.69% (*n* = 96) of the boys, while 11-year-olds represented 13.89% (*n* = 85). The 12-year-old girls accounted for 16.20% (*n* = 98), and 11-year-olds made up 12.73% (*n* = 77). The least represented age group for both boys and girls was the 17-year-olds, with only 0.49% (*n* = 3) for boys and 0.17% (*n* = 1) for girls.

#### Anthropometric values

The anthropometric characteristics, including height, weight and BMI for boys and girls across different age groups, are shown in Table 2. For boys, the average height ranged from 143.46 cm (± 7.08) at age 11 years to 162.03 cm (± 7.76) at age 16 years. Boys’ mean weight increased progressively with age, from 30.93 kg (± 5.15) at age 11 years to 45.56 kg (± 9.21) at age 16 years. Similarly, the average BMI for boys showed a gradual increase, from 15.22 kg/m^2^ (± 1.58) at age 11 years to 15.56 kg/m^2^ (± 1.56) at age 16 years. Overall, boys’ BMI remained relatively low across all age groups, staying below 17.0 kg/m^2^.

**TABLE 2:** Distribution of anthropometric values for all children living in rural areas of Ellisras according to age and sex, between March 2003 and April 2003, Limpopo province.

Age (years)	Boys				Girls			
	*n*	Height (cm)	Weight (kg)	BMI (kg/m^2^)	*n*	Height (cm)	Weight (kg)	BMI (kg/m^2^)
11	85	143.46 ± 7.08	30.93 ± 5.15	15.22 ± 1.58	77	142.31 ± 5.24	30.81 ± 4.33	16.53 ± 3.51
12	104	148.1 ± 7.29	34.59 ± 6.75	15.25 ± 1.77	98	146.93 ± 7.44	34.07 ± 6.99	16.25 ± 1.96
13	149	153.74 ± 6.65	38.81 ± 7.10	15.91 ± 1.69	129	154.19 ± 7.25	38.51 ± 7.03	16.89 ± 2.25
14	141	157.03 ± 7.60	41.12 ± 7.11	15.96 ± 2.03	152	158.17 ± 7.00	42.68 ± 7.40	14.49 ± 2.78
15	96	161.14 ± 7.56	44.65 ± 7.39	16.08 ± 1.59	118	161.31 ± 6.91	45.75 ± 7.45	17.62 ± 2.68
16	34	162.03 ± 7.76	45.56 ± 9.21	15.56 ± 1.56	30	160.27 ± 6.90	45.73 ± 8.07	17.89 ± 2.28
17	3	158 ± 11.27	39.67 ± 9.61	16.67 ± 2.10	1	159.00 ± 0.00	46 ± 0.00	15.2 ± 0.00

**Total**	**612**	**154.79 ± 7.89**	**39.33± 7.47**	**15.81 ± 1.76**	**605**	**154.60 ± 5.63**	**40.51 ± 5.90**	**16.41 ± 2.21**

BMI, body mass index.

For girls, the average height ranged from 142.31 cm (± 5.24) at age 11 years to 161.31 cm (± 6.91) at age 15 years. The mean weight for girls also followed an upward trend, from 30.81 kg (± 4.33) at age 11 years to 45.75 kg (± 7.45) at age 15 years. Girls’ BMI values ranged from 16.53 kg/m^2^ (± 3.51) at age 11 years to 17.62 kg/m^2^ (± 2.68) at age 15 years, with the highest mean BMI recorded at age 15 years.

In terms of overall averages, boys and girls had similar heights, with boys having a slightly greater average height (154.79 cm ± 7.89) compared to girls (154.60 cm ± 5.63). Regarding weight, girls had a slightly higher average weight (40.51 kg ± 5.90) than boys (39.33 kg ± 7.47). When considering BMI, girls had a higher average BMI (16.41 kg/m^2^ ± 2.21) compared to boys (15.81 kg/m^2^ ± 1.76).

At age 11 years, boys had a lower average BMI (15.22 kg/m^2^) than girls (16.53 kg/m^2^). However, from age 12 years onwards, the BMI values for boys and girls became more similar, with girls maintaining a slightly higher BMI. The highest BMI for boys was observed at age 15 years (16.08 kg/m^2^), while for girls, the highest BMI was also observed at age 15 years (17.62 kg/m^2^).

#### Anthropometric characteristics

The overall distribution of anthropometric characteristics for boys and girls is presented in Table 3. For boys, the mean height was 154.00 cm (± 9.35), with a range from 125 cm to 181 cm and a coefficient of variation (CV) of 6.08%.

**TABLE 3:** Anthropometric characteristics for all children living in rural areas of Ellisras according to sex, between March 2003 and April 2003, Limpopo province.

Variable	Boys			Girls		
	Mean ± s.d	Range	CV (%)	Mean ± s.d	Range	CV (%)
Height (cm)	1.54 ± 9.35	125–181	6.08	1.54.2 ± 9.42	122–178	6.11
Weight (kg)	38.82 ± 8.32	19–78	21.44	39.64 ± 8.66	21–80	21.86
BMI (kg/m^2^)	15.72 ± 1.78	11.2–29.4	11.32	17.05 ± 2.66	10.9–29.4	15.6

s.d., standard deviation; CV, coefficient of variation.

The average weight for boys was 38.82 kg (± 8.32), ranging from 19 kg to 78 kg, and the CV for weight was 21.44%. The mean BMI for boys was 15.72 kg/m^2^ (± 1.78), with values ranging from 11.2 kg/m^2^ to 29.4 kg/m^2^ and a CV of 11.32%.

For girls, the mean height was 154.20 cm (± 9.42), with a range from 122 cm to 178 cm and a CV of 6.11%. The average weight for girls was 39.64 kg (± 8.66), ranging from 21 kg to 80 kg, with a CV of 21.86%. The mean BMI for girls was 17.05 kg/m^2^ (± 2.66), with values ranging from 10.9 kg/m^2^ to 29.4 kg/m^2^ and a CV for BMI of 15.6%.

### Prevalence of weight status

The prevalence of different weight statuses for the entire sample, as well as by sex, is summarised in Table 4. A total of 525 children (43.10%) were classified as underweight, with a high proportion of boys (64.57%, *n* = 339) compared to girls (35.43%, *n* = 186). For normal weight, 682 children (56%) were classified as having a healthy weight, with 39.74% (*n* = 271) of them being boys and 60.26% (*n* = 411) being girls. The prevalence of overweight was very low, with only 5 children (0.41%) categorised as overweight, including 1 boy (20%) and 4 girls (80%). Similarly, 5 children (0.41%) were classified as obese, with 1 boy (20%) and 4 girls (80%) in this category.

**TABLE 4:** Prevalence of weight status for all children living in rural areas of Ellisras between March 2003 and April 2003 according to sex, Limpopo province.

Variable	All		Boys		Girls	
	*n*	%	*n*	%	*n*	%
Underweight	525	43.10	339	64.57	186	35.43
Normal weight	682	56.00	271	39.74	411	60.26
Overweight	5	0.41	1	20.00	4	80.00
Obesity	5	0.41	1	20.00	4	80.00

### Inferential statistics

#### Predictors of obesity

The results of the multivariate logistic regression analysis examining the influence of age and sex on obesity risk are presented in Table 5. The multivariate logistic regression analysis revealed that individuals aged 13–14 years had 1.45 times higher odds of obesity compared to the reference group (age 11 years), with an adjusted odds ratio (AOR) of 1.45 (95% CI: 0.90–2.33) and a statistically significant result (*p* = 0.03). Among those aged 15 years, the odds of obesity were 2.10 times higher compared to 11-year-olds, with an AOR of 2.10 (95% CI: 1.35–3.25) and a highly significant result (*p* < 0.001). Additionally, sex played a significant role, with females having 2.8 times higher odds of obesity than males, with an AOR of 2.80 (95% CI: 1.60–5.01) and a highly significant result (*p* < 0.001). These findings indicated that both age, particularly during the adolescent years (13–15 years), and female sex were significant factors associated with a higher risk of obesity in this population.

**TABLE 5:** Multivariate logistic regression analysis examining the influence of age and sex on obesity risk.

Variable	AOR	95% CI	*P*
**Age (years)**			
11	-	-	-
12	1.25	0.95–1.65	0.12
13	1.45	1.05–2.03	0.03
14	1.75	1.25–2.55	0.001
15	2.1	1.40–3.15	< 0.001
16	1.55	0.80–2.97	0.18
17	1.8	0.85–3.70	0.12
**Sex**			
Male	-	-	-
Female	2.8	1.60–5.01	< 0.001

AOR, adjusted odds ratio; CI, confidence interval.

## Discussion

The current study investigated the nutritional status of children residing in rural areas of Ellisras, using BMI cut-off points from the CDC reference standard, between March and April 2003. The study found that the majority of participants were in the middle adolescent age range of 13–15 years, reflecting trends observed in rural South Africa where children in this age group are more likely to be enrolled in school and accessible for health and educational studies. The higher representation of adolescents aged 13–15 years is likely because of their school attendance, as this age group is typically still in primary or secondary education, making them prime candidates for health interventions and community initiatives.

Conversely, the study found low representation of 11-year-olds and 17-year-olds. Overall, the age distribution showed a higher proportion of participants in the middle adolescent years (ages 13–15 years), with 14-year-olds being the largest subgroup among girls and 13-year-olds the largest subgroup among boys. The smallest groups were found at both ends of the age spectrum, with fewer 11-year-olds and 17-year-olds. The small number of 11-year-olds could be as a result of them being in early education or not yet subject to compulsory schooling, while the low participation of 17-year-olds (who made up only 0.49% of boys and 0.17% of girls) likely reflects migration patterns, where adolescents in this age group leave rural areas for further education or employment in urban centres. This trend is consistent with other rural health studies in South Africa.

These findings align with previous research, such as studies by Fields GS,^17^ which also show that middle adolescents (ages 12–15 years) are the most prevalent in rural health studies. In contrast, urban studies often report a broader age range, with greater representation of older adolescents (ages 16–17 years), because of better access to education and higher retention rates in urban areas.^15,16^

The anthropometric data for boys and girls observed in this study revealed consistent trends in height, weight and BMI across different age groups. For boys, both height and weight increased progressively with age, reflecting typical growth patterns during adolescence. Their BMI remained relatively low across all age groups, with the highest value recorded at age 15 years. This could reflect the nutritional patterns and growth trajectories in rural South African communities, where children may experience both undernutrition and lower levels of access to caloric foods, especially in early childhood. The relatively low BMI across age groups suggests that these children were generally undernourished, with body mass increasing as they reached adolescence, potentially because of changing dietary habits, increased access to food or different metabolic demands during puberty.^18^

Comparatively, studies in more urbanised or developed settings tend to show a different pattern, particularly in higher income countries or urbanised areas. For example, research from a study of American children found that BMI typically rises consistently across childhood and adolescence, with higher rates of overweight and obesity because of greater access to high-calorie diets, sedentary lifestyles and more processed foods,^19^ for example, childhood BMI and the risk of coronary heart disease in adulthood. Another study observed higher BMI trajectories from early childhood into adulthood, indicating that nutrition and lifestyle factors in urban settings tend to lead to higher rates of obesity.^20^

Additionally, a study in South Africa found that rural children typically showed lower BMI compared to urban counterparts, which aligns with the findings of the Ellisras Growth Study.^2^ The study indicated that rural children are more likely to experience stunting and underweight issues because of food insecurity, limited access to diverse and nutritious food and lower socio-economic conditions. This research reinforces the idea that lower BMI values are more commonly found in rural areas, especially when compared to urbanised populations, where greater access to food and nutrition can lead to higher BMI values and greater risks for obesity.

For girls, height followed a similar upward trend, and weight also increased steadily across the adolescent years. Girls consistently had higher BMI values than boys, especially from age 11 onwards. This is consistent with existing research, which suggests that girls tend to have higher BMI during adolescence, likely because of the earlier onset of puberty and differences in fat distribution compared to boys.^21^

In terms of overall averages, boys and girls had similar heights, although boys were slightly taller. However, girls had a higher average weight and BMI, aligning with studies that report greater fat accumulation in girls during puberty because of hormonal changes.^22^ At age 11 years, boys had a lower BMI compared to girls, as girls typically accumulate more body fat at this stage, while boys are still undergoing rapid linear growth. From age 12 years onwards, the BMI values for both sexes became more similar, with girls maintaining a slightly higher BMI across all age groups. This shift reflects the onset of puberty, during which boys gain muscle mass and girls continue to accumulate fat.^21^

Both boys and girls experienced their highest BMI at age 15 years, a finding consistent with other studies suggesting that this is the period when puberty concludes and fat mass tends to stabilise.^23^ This study’s results mirror these trends, confirming the expected patterns of growth, development and fat accumulation during adolescence, with distinct gender differences in BMI development.^24^

Studies conducted in South Africa have highlighted a rising prevalence of both underweight and obesity, particularly in rural areas. Another study found that 43.4% of rural South African children were underweight, while obesity rates were climbing in urban areas because of lifestyle changes and a shift towards high-calorie, low-nutrient foods.^25^ This dual burden of malnutrition has emerged as a significant public health issue across the country. Additionally, another study reported that 9.1% of South African children in rural areas were overweight or obese, with urban children exhibiting even higher rates.^26^ These findings reflect the increasing coexistence of undernutrition and rising obesity rates in both rural and urban populations.

In Kenya, a study revealed that 47.3% of rural children were underweight, while 5.3% were overweight or obese.^27^ Similar to other sub-Saharan African countries, rural Kenyan children face a dual burden of malnutrition, with undernutrition remaining a major concern despite the rising rates of obesity in urban areas. The increase in childhood obesity in Kenya has been linked to urbanisation, dietary shifts towards processed foods and reduced physical activity.

In Nigeria, a study found that 30.4% of rural children were underweight, while 12.2% were overweight or obese.^28^ The study emphasised that while undernutrition continues to be a significant issue, obesity is on the rise, particularly among urban children. However, rural children are not immune to these changes, and childhood obesity is increasingly becoming a public health concern in rural areas as well.

Globally, the World Health Organization (WHO) reports that approximately 45% of children in low-income countries, including those in sub-Saharan Africa, experience some form of malnutrition, such as underweight, stunting or overweight.^29^ The prevalence of obesity is notably increasing in urban areas of developing countries, including parts of sub-Saharan Africa, where rapid urbanisation, increased consumption of high-calorie foods and sedentary lifestyles are contributing to the rising obesity rates among children and adolescents. In Latin America, for example, studies have shown that 11.7% of children in Brazil and 9.8% in Mexico are classified as overweight or obese, with urban populations exhibiting much higher rates.^30^ These global trends align with those observed in South Africa and Kenya, where the shift from rural to urban living has brought about changes in diet and activity levels, further contributing to the rising burden of obesity.

The multivariate regression analysis conducted in this study identified key age- and sex-related factors that influence the odds of obesity among adolescents. The results revealed that adolescents aged 15–16 years had significantly higher odds of obesity compared to those aged 11–12 years. This could be because of various physiological and behavioural factors associated with puberty, including hormonal changes, changes in metabolism and shifts in physical activity and dietary habits.^15^ This result is consistent with findings from other countries in sub-Saharan Africa. For instance, a study reported that the prevalence of obesity among South African children increases with age, particularly during adolescence, as dietary habits and lifestyle behaviours shift.^31^

Similarly, female adolescents exhibited significantly higher odds of obesity than their male counterparts. Several factors may contribute to this difference, including hormonal changes during puberty that lead to increased fat deposition in females, as well as differences in physical activity levels, dietary habits and societal pressures regarding body image.^32^ This pattern has been reported globally, particularly in low- and middle-income countries where the prevalence of obesity is rising in both rural and urban populations, but with more marked increases in females.

The study focused on the same group of children measured in November 1999 as part of the Ellisras Longitudinal Growth Study.^33^ This research specifically examined the relationship between BMI and dietary intake in secondary school children from a rural South African area.

The findings of this study were important for understanding how nutritional intake influences the growth and health of children, particularly in rural settings. By analysing the dietary habits and BMI of these children, the study revealed key insights into the nutritional challenges faced by rural populations. The research highlighted the potential connection between undernutrition and a lack of sufficient or balanced food intake, as well as how overnutrition (e.g. excessive calorie consumption) can also be a concern, especially in the context of rapid lifestyle changes.

### Strengths and weaknesses of the study

The study’s strengths lie in its large sample size and data drawn from 42 rural settlements; the study offers a broad and diverse representation of children in rural Ellisras, providing valuable insights into how weight status and obesity risk differ between boys and girls at various stages of adolescence. This dual analysis of age and sex enables a better understanding of gender-based health disparities within the rural population. However, the study’s cross-sectional design presents a limitation, as it only offers a snapshot of the population at a single point in time, preventing the establishment of causal relationships between age, sex and obesity risk. Consequently, while the study contributes valuable information on rural health issues in Ellisras, its findings may not be generalisable to urban areas or other regions of South Africa or beyond. Urban–rural disparities in health outcomes can vary significantly, and the trends observed in rural Ellisras may not reflect those in other populations. Another limitation of the present study is the absence of key confounding variables such as physical activity levels, socio-economic status and parental education. These factors are known to influence nutritional status and obesity risk, and their exclusion may have affected the interpretation of our findings. Without adjusting for these confounders, the associations observed between anthropometric measures and nutritional status may not fully account for external influences.

### Recommendations

The findings of this study highlight the need for targeted, age- and sex-specific interventions to address the dual challenges of undernutrition and obesity in rural children in Ellisras. By focusing on both nutritional improvements and obesity prevention, with special attention to gender differences and the specific needs of children at various stages of adolescence, public health initiatives can help improve the overall health and well-being of children in rural Limpopo province. The following recommendations can serve as a blueprint for developing effective, community-based health interventions that support the healthy growth of children in similar rural settings:

- As the study found a higher prevalence of underweight among boys (64.57%), targeted nutritional interventions should be designed to address this issue, particularly for boys in rural areas. Programmes could focus on improving access to balanced diets rich in calories, protein and micronutrients to support healthy growth.
- Launch community-based nutrition education programmes that emphasise the importance of balanced diets, particularly for children aged 11–14 years, who were identified as being at risk of undernutrition. Local health workers could play a key role in delivering these interventions.
- The study found that girls had significantly higher odds of obesity compared to boys, particularly in the age group of 13–15 years. Obesity prevention programmes should be specifically tailored to this group, addressing lifestyle factors such as diet, physical activity and sedentary behaviour.
- Initiatives that encourage physical activity and reduce sedentary time (e.g. through school-based sports programmes, community exercise activities and promoting walking or cycling) should be promoted, especially among girls who may be more prone to weight gain during puberty.
- Schools and community centres could implement educational campaigns about the risks of sugary foods, unhealthy snacks and processed foods, while promoting locally available, nutritious alternatives.

## Conclusion

The results of the current study highlight the importance of addressing both underweight and obesity among children in rural Ellisras, with particular attention to the higher obesity risk in girls and the greater prevalence of underweight in boys. The study also suggests that girls in this rural population are more at risk of obesity than boys, especially during mid-adolescence (ages 13–15 years). The higher BMI in girls, combined with the higher odds of obesity in females, suggests that interventions targeting obesity prevention may need to be more focused on girls in this age group.^34^ The high prevalence of underweight children, particularly among boys, points to a significant nutritional issue in the rural areas of Ellisras. Addressing undernutrition and ensuring access to proper nutrition for all children, especially boys, should be a priority.

The increase in obesity risk with age, particularly in the 15-year-olds, suggests that preventive measures should begin early in adolescence to address the risk factors associated with obesity, such as diet, physical activity and education about healthy lifestyles. Public health interventions should consider sex and age as key factors when designing strategies to combat undernutrition and obesity. Policies aimed at improving nutrition, promoting physical activity and addressing gender-specific factors affecting weight should be implemented in rural areas to improve overall child health outcomes.
